# Serum CD203c+ Extracellular Vesicle Serves as a Novel Diagnostic and Prognostic Biomarker for Succinylated Gelatin Induced Perioperative Hypersensitive Reaction

**DOI:** 10.3389/fimmu.2021.732209

**Published:** 2021-09-28

**Authors:** Zheng Qi, Qiong Xue, Haitao Wang, Bin Cao, Yu Su, Qinghe Xing, Jian-Jun Yang

**Affiliations:** ^1^ Department of Anesthesiology, Pain and Perioperative Medicine, The First Affiliated Hospital of Zhengzhou University, Zhengzhou, China; ^2^ Institutes of Biomedical Sciences, Fudan University, Shanghai, China

**Keywords:** perioperative hypersensitive reaction, extracellular vesicle, mast cells, basophils, biomarker

## Abstract

**Background:**

Perioperative hypersensitivity reaction (HR) is an IgE-FcϵRI-mediated hypersensitivity reaction with degranulation and activation of mast cells and basophils. Several studies have focused on assessing the degranulation and activation of mast cells and basophils to diagnose and predict the prognosis of drug induced HR. However, it is challenging to isolate sufficiently pure mast cells and basophils from human sources to investigate. Effective biomarkers to assess mast cells and basophils activation *in vivo* could potentially have high diagnostic and prognostic values. In the present study, we investigated EVs pelleted from serum in patients with succinylated gelatin induced HR.

**Methods:**

Extracellular vesicles (EVs) were isolated using a total exosome isolation kit and ultracentrifugation, characterized by Western blot, transmission electron microscopy, and nanoparticle tracking analysis. Basophils were isolated from fresh peripheral blood by negative selection using Basophil Isolation Kit II. Human mast cell line was stimulated with IL4. The expression levels of proteins related to the hypersensitive response were evaluated by Western blotting and flow Cytometer. Histamine and tryptase levels were tested using a commercial ELISA kit, and gene expression of inflammatory mediators was evaluated by qRT-PCR. The receiver operating characteristic (ROC) curve was used to evaluate the specificity and sensitivity of biomarker in predicting HR.

**Results:**

The concentration of EVs and protein expression level of CD63, FcϵRI, CD203c and tryptase were significantly (*p*< 0.05) increased in HR samples. The expression level of mast cell/basophil specific CD203c were significantly increased in EVs derived from serum and basophils of HR patients, and the CD203c^+^-EVs production in mast cells is dramatically increased in the presence of IL4, which positively correlated with histamine, tryptase and inflammatory mediators. Moreover, the ROC curve of EVs concentration and CD203c expression indicated that CD203c^+^-EVs had a strong diagnostic ability for HR.

**Conclusion:**

Serum CD203c+-EVs serves as a novel diagnostic and prognostic biomarker for HR.

## Introduction

Perioperative hypersensitivity reaction (HR) is caused by a rapid hypersensitivity reaction triggered by perioperative drugs (usually anesthetics) that affects multiple tissues, organs, and systems throughout the body and poses a serious threat to the lives of surgical patients. Common perioperative drugs that trigger HR include Neuromuscular Blocking Agents (NMBA), antibiotics, Succinylated Gelatin (SG), blood products, etc. (1). The diagnosis of drug induced HR is difficult because a wide range of different drugs (or metabolites) can trigger immune- and non-immune-mediated pathologies with different and sometimes unclear pathomechanisms. HR is an IgE-FcϵRI-mediated hypersensitivity reaction with degranulation and activation of mast cells and basophils (2), which release mediators, including histamine, tryptase and inflammatory chemokines (3, 4). Based on the knowledge of mast cells and basophils, several studies have focused on assessing the degranulation and activation of mast cells and basophils to diagnose and predict the prognosis of drug induced HR. However, it is challenging to isolate sufficiently pure mast cells and basophils from human sources to study them. Therefore, studies have been focused on exploring potential biomarkers of mast cell and basophil activation *in vivo*. Histamine, lipid mediators and tryptase have been reported as common biomarkers of mast cells and basophils (5–7). In addition, CD203c and CD63 have been considered as specific surface biomarkers and used to assess degranulation and activation of mast cells and basophils (8–11). However, different practical and technical shortcomings limit its validation and clinical application (12, 13), moreover, the sensitivity and specificity of inflammatory mediators for diagnosis HR is not satisfactory. Therefore, effective biomarkers to assess mast cell and basophil activation *in vivo* could potentially have high diagnostic and prognostic values, and the availability of a sensitive, specific, rapid, and reliable test to diagnose HR would be more than welcome.

Extracellular vesicles (EVs) are a type of membrane-encapsulated vesicles released from cells, biofluids, and tissues into the extracellular space and contain miRNAs, DNA, mRNAs, and proteins (14). EVs have characteristic surface tetraspanin proteins, for example, CD9, CD63, and CD81. Although there are typical proteins on the surface or inside of EVs (e.g., tetraspanins, cytoskeletal proteins, and chaperones), the actual content(miRNAs, DNA, mRNAs, and proteins) varies depending on the cellular source, so it is expected that EVs from different cells contain specific content which lead to different effects in other target cells and tissues. EVs have been reported to play a physiological role in modulating immune responses. Recent evidence showed that EVs derived from activated mast cells could be uptaken by resting and/or other activated mast cells through endocytosis, and subsequently triggers mast cells degranulation and inflammatory mediators production (15–17). Moreover, Carroll-Portillo et al. and Rosa Molfetta et al. demonstrated that EVs derived from mast cells contain FcϵRI, which represents a potential amplification mechanism in which the antigen is recovered for continued cross-linking (18, 19). Recent studies have shown that EVs play a critical role in the diagnosis and prediction of diseases (20–22). In addition, EVs have been reported to modulate immune responses by regulating gene expression (23), transporting cytokines (24) and nucleic acids such as proinflammatory microRNAs (25).

In the present study, we examined the biomarkers expression levels on the surface of EVs derived serum and basophils from SG induced HR patients, and human mast cells(HMCs) stimulated with IL4. As a result, we first found that the expression level of mast cell/basophil specific biomarker CD203c were significantly increased in EVs derived from serum and basophils of HR patients, and the CD203c^+^-EVs production in mast cells is dramatically increased in the presence of IL4, which positively correlated with histamine, tryptase and inflammatory mediators. Moreover, the level of CD203c^+^-EVs showed a satisfactory sensitive and specific response for HR and treatment. Our study may provide a novel diagnostic and prognostic biomarker for HR.

## Materials And Methods

### Clinical Sample Collection

The use of human material was approved by the local ethics committee (reference number: 2021- KY -0378-002, The First Affiliated Hospital of Zhengzhou University) and written informed consent was obtained from all patients. All patients with HR had a clinical syndrome of marked hypotension and/or urticarial rash, which was immediately treated with epinephrine,vasopressors, antihistamines and glucocorticoids. HR patients were diagnosed according to Ring and Messmer severity scale (26). HR Serum samples (grades III-IV Ring and Messmer classification) were obtained from 20 patients (mean age: 49.3 ± 8.7 years, range: 38-68 years) with treatment at 0H (HR -0H), 2H (HR -Treated-2H) and 24H (HR -Treated-24H) after initiation of drug treatment HR. Control serum samples were obtained from 20patients without HR (mean age: 56.7 ± 8.0 years, range: 41-71 years). Fresh blood samples were collected in Vacutainer tubes, and serum was collected by coagulation at room temperature for 1 h and centrifugation at 1500 ×g for 10 min at 4°C.

### Human Serum-Derived EVs Isolation and Characterization

EVs were isolated from 500μL serum using a total exosome isolation kit (#4478360, Thermo Fisher Scientific, Waltham, MA, USA) according to the manufacturer’s instructions. Briefly, we centrifuged 500μl serum samples at 2000 × g for 30 minutes to remove cells and debris, then transferred the supernatant to a new tube, followed by adding 100μl the Total Exosome Isolation reagent and incubated in 4° for 30min. Subsequently centrifuged the samples at 10,000 × g for 10 minutes at room temperature to pellet the EVs, then the pellet was resuspended in PBS and stored at -80°C for use in the following experiments.

Differential centrifugation and ultracentrifugation steps were applied to pellet EVs from conditional supernatant according to protocols previously published (27). Briefly, we applied sequential centrifugation steps for 10 min at 300xg to remove cells, followed by 10 min at 2,000xg to remove dead cells and finally for 30 min at 10,000xg to remove cell debris. This procedure was followed by ultra-centrifugation of the supernatant at 120,000xg for 70 min at 4°C and subsequently dispersing the pellet in PBS, after which the solution was filtered through a 0.22 µm filter. Finally, the EVs were pelleted at 120,000xg for 70 min at 4°C and the pellet was resuspended in 200μl PBS and stored at -80°C for use in the experiments.

EVs were visualized by transmission electron microscopy (TEM). For negative staining, 20 μl EVs was added onto parafilm and a formvar (polyvinyl formal)-carbon coated 400 copper mesh grid was placed on top of the fluid for 10 min at RT. Then the grid was incubated with 2% phosphotungstic acid for 1 min and dried at RT for 10 min. EVs were investigated with an acceleration voltage of 100 kV and 100,000 x magnification.

The concentration and size distribution of EVs were determined by nanoparticle tracking analysis (NTA; NS300, Malvern Instruments, Malvern, UK). Accuracy of NTA was confirmed with 100 nm polystyrene beads (Sigma-Aldrich, Germany) immediately before measurements. EVs samples were diluted 1:50 in PBS and measurements performed at 25°C, five measurements of 30 second were recorded for each EVs sample. The classical surface markers (CD9, CD63, CD81) of EVs were verified by western blotting.

### RNA Extraction and Quantitative Real-Time Polymerase Chain Reaction (qRT-PCR) Analysis

Total RNA from serum was isolated using Absolutely RNA Miniprep Kit (Agilent Technologies, Santa Clara, CA, USA) according to the manufacturer’s instructions and then synthesized using a cDNA synthesis kit (Agilent Technologies). RNA concentration and quality were determined using a NanoDrop™ (ND -1000 spectrophotometer, Thermo Fisher Scientific). Relative mRNA levels were determined using Brilliant III Ultra-Fast SYBR^®^ Green QPCR Master Mix (Agilent Technologies) on an MX3005P QPCR system (Agilent Technologies). All genes were relatively analyzed, calibrated to control group expression, and normalized to that of GAPDH and 18S. All qPCR experiments in this study were performed in duplicates containing 20-50 ng of cDNA. The sequences of all primers used for qRT-PCR in this study were as follows: *IL1B*: 5’-TGCCACCTTTTGACAGTGATGA-3’ (forward), 5’-TGTGCTGCTGCGAGATTTGA-3’ (reverse); *IL4*: 5’-GAAAAAGGGACTCCATGCAC-3’ (forward), 5’- TCTTCAAGCACGGAGGTACA-3’ (reverse); *IL13*: 5’- GCAATGGCAGCATGGTATGG-3’ (forward), 5’-CCCGCCTACCCAAGACATTT-3’ (reverse); *IL33*: 5’- CAGGCCTTCTTCGTCCTTCAC-3’ (forward), 5’- TCTCCTCCACTAGAGCCAGCTG’ (reverse); *GAPDH*: 5’-GTGAAGGTCGGTGTGAACG-3’ (forward), 5’-AATCTCCACTTTGCCACTGC-3′(reverse); *18S*: 5′-AAACGGCTACCACATCCAAG-3’ (forward); 5’-CCTCCAATGGATCCTCGTTA-3’ (reverse).

### Protein Extraction and Western Blotting Analysis

Serum proteins were extracted using a phosphatase-containing serum protein extraction kit (BestBio, Shanghai, China). First, 400μl reagent was added into 200μl serum, mixed thoroughly and incubated 5min at 4°, then centrifuged at 14000g for 10min at 4° and removed the supernatant to a new cold 1.5ml tube, ready for BCA test or following experiments. EVs were quantified using a BCA protein kit assay (Thermo Fisher Scientific). An equivalent amount of cell lysates, serum protein, or EVs were mixed with SDS-sample loading buffer, followed by boiling for 5 min at 95°C, and subjected to 12% SDS -PAGE. After electrophoretic separation, the proteins were transferred onto a 0.22-µm PVDF membrane, then blot membranes were first stained with Ponceau Red solution (loading control), washed and then blocked with 5% BSA in TBST for 1 h at room temperature. Membranes were then incubated with primary antibodies, CD9 (ThermoFisher 10626D) 1:1000, CD81 (ThermoFisher 10630D) 1:1000, CD 63 (ThermoFisher 10628D) 1:1000, CD203c (Cell signaling 71414) 1:1000, FcϵRI(Abcam ab229889) 1:500, Tryptase (Cell signaling 51550) 1:1000 at 4°C overnight, followed by goat anti-rabbit IgG secondary antibody (Jackson Immuno Research) for 1 h at room temperature. Protein bands were visualized using ECL detection reagents (Thermo Scientific). Image J software (1.8.0, National Institutes of Health, Bethesda, MD, USA) was used to quantify the density of bands and normalize them to the control group.

### Histamine and Tryptase Detection in Serum

The concentration of histamine and tryptase in serum was determined using a commercial Enzyme-Linked Immunosorbent Assay (ELISA) kit(#SEB070Hu, Cloud-Clone, Wuhan, China), following the manufacturer’s instructions.

### Basophils Isolation and EVs Preparation

Basophils were isolated from fresh peripheral blood of 5 healthy donors and 5 HR patients by negative selection using Basophil Isolation Kit II (Miltenyi Biotec, Paris, France) and cytoflex (Beckman Coulter, USA). Peripheral blood mononuclear cells (PBMCs) were isolated using Ficoll density gradient(LDS1075, Tianjin, China). First, non-basophil cells in PBMCs were labeled with biotin-conjugated antibodies(CD3, CD4, CD7, CD14, CD15,CD16, CD36, CD45RA, HLA-DR and CD235a) and anti-biotin microbeads, then non-basophils were depleted by a BeyoMag™ separator(FMS012, Beyotime, China). In the second step, the basophils were labeled with CD123 microbeads, subsequently basophils were eluted from column after magnetic separation. The purity of the basophils as determined by flow cytometry, the expression of CD123 was over 95%.

2x10^6^ Basophils were seeded in T75 flask with serum free x-vivo 15 medium (Lonza). After 48h incubation, conditional culture supernatant was collected for EVs isolation using ultracentrifugation (2.2).

### Mast Cells Stimulation and EVs Preparation

2x10^6^ Human mast cells (HMCs, #CP-H110, Procell, China) were seeded in T75 flask with serum free x-vivo 15 medium (Lonza). After HMCs reached 70%-80% confluence, changed medium supplied with recombinant human IL4 protein (#6507-IL/CF, R&D, USA) 2ng/ml, 5ng/ml and 10ng/ml. After additional 48 incubation, conditional supernatant was harvest for EVs isolation using ultracentrifugation (2.2).

### BrdU Incorporation Assay

A BrdU–ELISA (enzyme-linked immunosorbent assay)-based assay (Roche, Penzberg, Germany) was performed to determine cell proliferation according to the manufacturer’s protocol. 5 x 10^3^ HMCs were seeded into 96-well plates. After 24h culturing, culture serum free x-vivo 15 medium (Lonza) was exchanged to medium containing BrdU and differential concentration (2ng/ml, 5ng/ml and 10ng/ml) of IL4. After an additional 24 h, the amount of BrdU incorporated into the cells was determined by binding of a mouse anti-BrdU antibody conjugated with horseradish peroxidase. After colour development the signal was monitored at 450/690 nm with a MULTISKAN FC ELISA reader (Thermo, USA).

### Flow Cytometer for EVs Specific Markers

Flow cytometry was performed to evaluate expression levels of mast cell/basophil specific markers on the surface of EVs, following the protocol described by Théry et al (27), with modifications. 10μg (measured by BCA assay) EVs from control and HR groups were incubated with 10 μl latex beads(latex beads without EVs as negative control) at room temperature for 20min, in a 1.5-ml microcentrifuge tube. Subsequently, the EVs/beads complexes were incubated with primary antibodies against CD63 (FITC-conjugated, #353005, Biolegend); CD203c (PE-conjugated, #324605, Biolegend) and FcϵRI (PE-conjugated, #334619, Biolegend) diluted in PBS/0.5% BSA 1h at 4°. Wash twice with PBS/0.5% BSA, resuspend in 200 μl PBS/0.5% BSA and then subject to cytoflex flow cytometry(Beckman Coulter, USA).

### Statistical Analysis

All data are expressed as mean ± standard deviation (SD). Statistical analysis was performed using Prism 8.02 software (GraphPad Software, USA). Comparisons between two groups were analyzed by independent two-tailed Student’s t-tests, and comparisons between more than two groups were analyzed by one-way analysis of variance (ANOVA) with Newman-Keuls multiple comparison test. Each assay was performed with at least three independent experiments. *p*< 0.05 was considered statistically significant.

## Results

### Characterization of EVs Derived From Control and HR Serum

**Figure 1:**
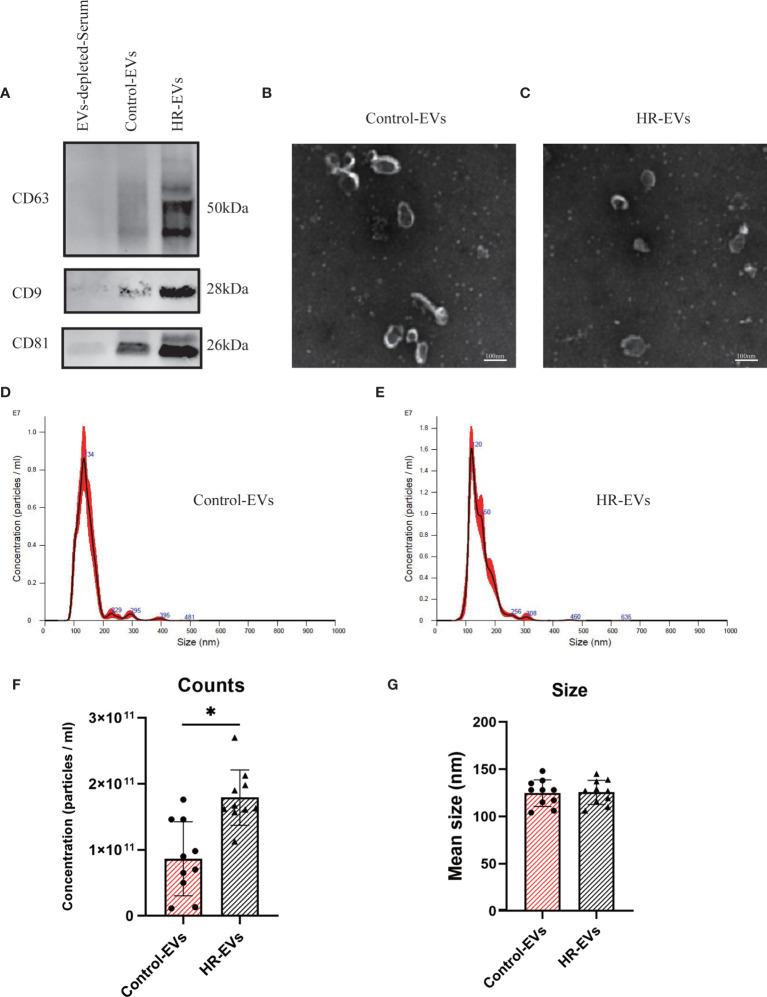
Characterization of control EVs and HR-EVs. EVs were processed for western blot, TEM and NTA after isolated from 500μl serum of control and HR groups. **(A)** The same amount (30μl) of control EVs, HR-EVs and EVs-depleted-Serum were identified using western blot. Representative western blot image showing bands of standard surface markers (CD9, CD63, CD81) of control EVs and HR-EVs. **(B, C)** Morphology of control EVs and HR-EVs was monitored by TEM; Scale bar: 100 nm. **(D, E)** Particle size distribution of control EVs and HR-EVs was determined by NTA. Distribution of EVs with a size of 90-200 nm in diameter in both groups. **(F, G)** Quantitative comparison between control EVs and HR-EVs in count and size measured by NTA; n = 10; All values represent mean ± standard deviation. *p < 0,05, independent two-tailed Student’s *t*-tests.

To validate the EVs in the control and HR groups, Western blotting was performed to verify the classical biomarkers (CD9, CD63 and CD81) of the EVs (**Figure 1A**) after EVs isolation. EVs-depleted serum served as a negative control. EVs morphology was monitored by TEM (**Figures 1B, C**), and particle size distribution and concentration were tested by NTA (**Figures 1D, E**). The results of TEM and NTA indicated the average size of both EVs samples to be approximately 120 nm, which was consistent with that of EVs  (28). These results demonstrate that the vesicles obtained and analyzed are EVs.

### HR Promoted the Concentration of CD63^+^-EVs and CD203c^+^-EVs Derived From Serum

To examine the concentration difference between EVs in the control and HR groups, NTA was used to determine the concentration of EVs pelleted from the same volume(500μl) of serum. Quantitative comparison of counts showed that the concentration of EVs was significantly increased in the HR groups, but there was no significant difference in size between the control and HR groups (**Figures 1F, G**). These results suggest that HR promotes the production of EVs.

**Figure 2:**
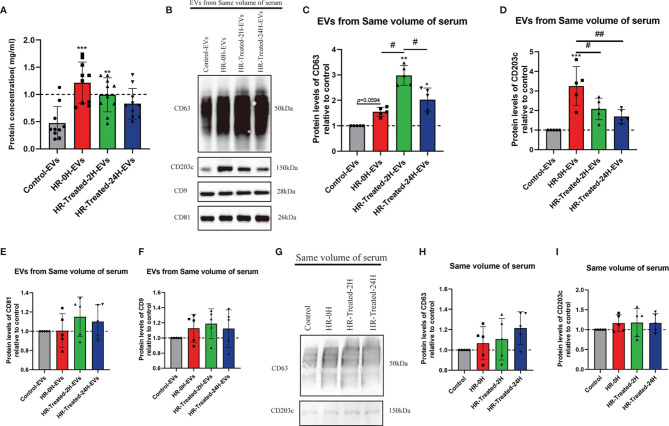
HR promoted the concentration of EVs and CD203c^+^-EVs and CD63^+^-EVs derived from serum. 500μl serum from control and different time point HR groups were used to pellet EVs, finally, the EVs were resuspended in 200μl PBS. The total concentration of EVs was determined by a BCA assay. The protein expression levels of CD63, CD203c, CD9 and CD81 in EVs/serum were identified using western blotting. **(A)** Total concentration of EVs isolated from same volume serum of control and different time point HR groups, n = 10. **(B–F)** Relative CD63, CD203c, CD9 and CD81 protein expression levels of EVs derived from same volume of serum in control and HR groups, 30μl EVs/lane, n = 5 **(G–I)** Relative CD63 and CD203c expression levels in same volume of serum in the control and HR groups, 30μl serum/lane, n = 5. All values represent the mean ± standard deviation. Difference to control: **p < 0,01; ***p < 0,001; ^#^Difference between groups: ^#^ *p* < 0,05; ^##^ *p* < 0,01; 1 way ANOVA with Newman-Keuls Multiple Comparison Test.

To reveal the relationship between the concentration of EVs and HR, the BCA test was performed to test the protein concentration of EVs in the same volume of pelleted serum from control and different time points HR (and treated with epinephrine, vasopressors, antihistamines and glucocorticoids) groups. Our results showed that the concentration of EVs peaked at 0h and 2h after treatment and then decreased at 24h after treatment (**Figure 2A**).

We used Western blotting to assess the relative expression levels of CD63, CD203c, CD9, and CD81 of HR specific EVs. As shown in **Figures 2B–F**, the expression levels of CD63 and CD203c were significantly increased in the HR groups, and the expression level decreased 24 hours after treatment. On the other hand, the expression levels of CD9 and CD81 were increased in the HR groups, but no significant difference compared with the control group. These results suggest that HR promotes the concentration of CD63^+^- EVs and CD203c^+^- EVs.

We also determined the expression level of CD63 and CD203c in the same volume of serum. There was no significant difference between the control and HR groups (**Figures 2G** **–I**).

The above results indicate that the levels of CD63^+^-EVs and CD203c^+^-EVs were increased by HR and showed greater sensitivity to HR and treatment compared to serum.

### HR Increased the Mast Cell/Basophil Specific and HR Related Protein Expression Levels in EVs

**Figure 3:**
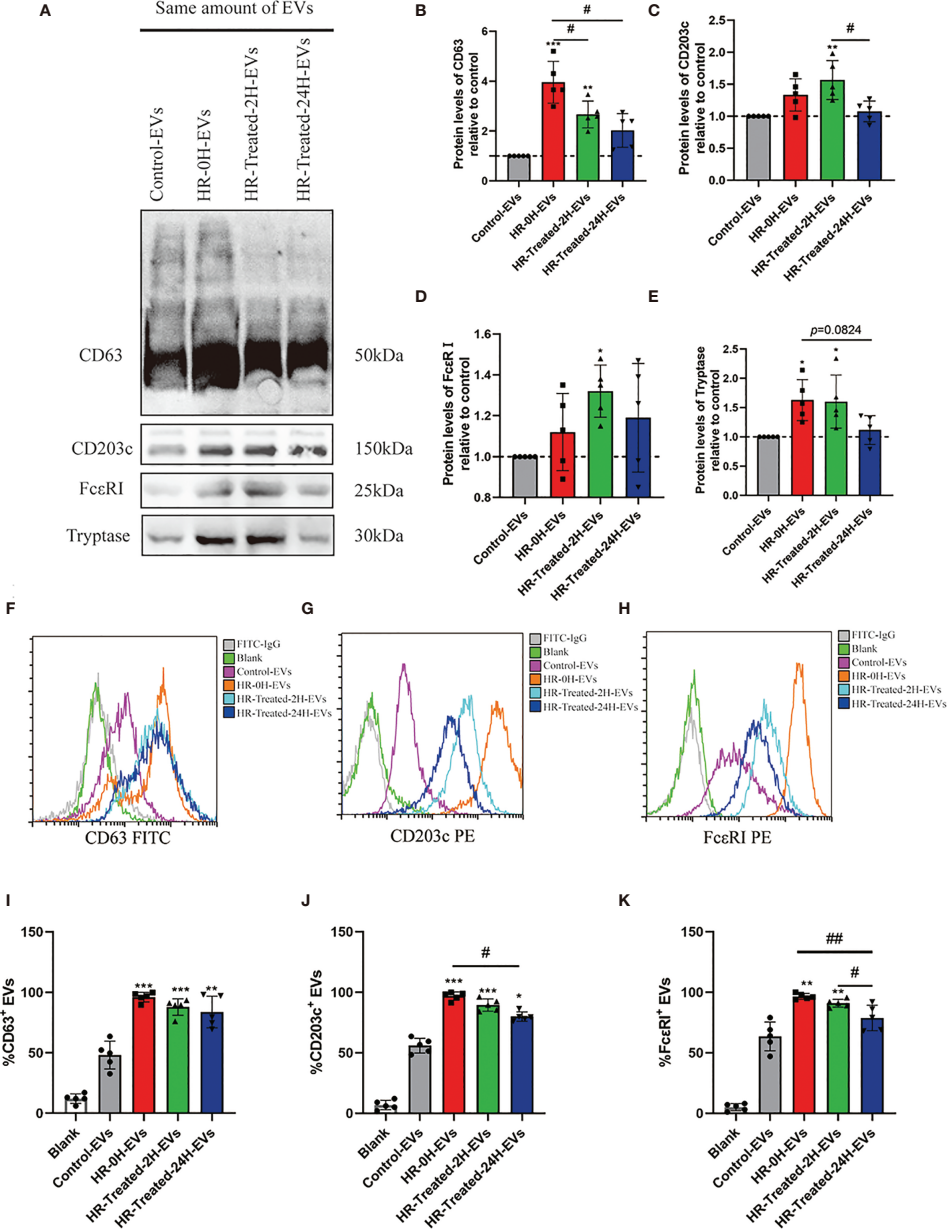
Mast cell/basophil specific protein expression levels in EVs. The same amount of EVs (20μg determined by BCA assay) in control and HR groups were used to detect the Mast cell/basophil specific protein (CD63, CD203c, FcϵRI and tryptase) expression levels by western blotting and Flow cytometry. **(A)** Western blotting was used to determine mast cell/basophil-specific protein expression in EVs from the control and HR groups. **(B–E)** Quantification of specific protein expression levels in different groups. The Control-EVs group is defined as control. **(F–H)** Analysis of the CD63, CD203c and FcϵRI expression on the surface of EVs in control and HR groups by flow cytometry with the indicated antibodies. Blank group (antibodies incubated latex beads without EVs) plays as a negative control. **(I–K)** Quantification of the percentage of CD63-positive, CD203c-positive, and FcϵRI-positive EVs in the control and HR groups. All values represent the mean ± standard deviation. Difference to control: *p < 0,05; **p < 0,01; ***p < 0,001; ^#^Difference between groups: ^#^ *p* < 0,05; ^##^ *p* < 0,01; 1 way ANOVA with Newman-Keuls Multiple Comparison Test; n = 5.

CD63 and CD203c have been reported as surface biomarkers of mast cells/basophils (29, 30), which were upregulated following aggregation of FcϵRI (8, 31). In this study, we use western blotting to evaluate the expression levels of CD63, CD203c and HR classical biomarkers (FcϵRI and tryptase) in EVs. As shown in **Figures 3A–C**, the expression of CD63 and CD203c significantly increased in the HR -0H and HR -treated-2H groups, and subsequently decreased in the HR -treated-24H group. Similarly, the expression levels of FcϵRI and tryptase were increased in the HR groups (**Figures 3D, E**). In addition, flow cytometry was performed to evaluate expression levels of FcϵRI, CD63 and CD203c on the surface of EVs in control and HR groups (**Figures 3F** **–H**). An analysis between groups was performed based on the percentage of EVs with positive expression of FcϵR I, CD63, and CD203c. At the onset of HR, the expression of CD63 on the surface of EVs was significantly increased and remained at a high level after treatment(**Figure 3I**); The expression of CD203c and FcϵRI on the surface of EVs was upregulated onset of HR, but decreased after 24h treatment (**Figures 3J, K**). Our results indicated that the expression levels of FcϵRI, CD63 and CD203c were significantly upregulated in HR groups, and the expression of CD203c and FcϵRI showed sensitivity to treatment after 24h. Furthermore, the expression levels of CD203c in EVs of other drug(Neuromuscular Blocking Agents, Succinylated Gelatin and Antibiotics) induced HR were determined (**Figure S1**). Similarly, the percentage of CD203c^+^-EVs was significantly increased in HR groups. These results suggest that the expression level of CD203c in EVs probably plays as a novel biomarker for HR.

### Proliferation of HMCs and CD203c^+^-EVs Production Were Promoted After Treated With IL4

**Figure 4:**
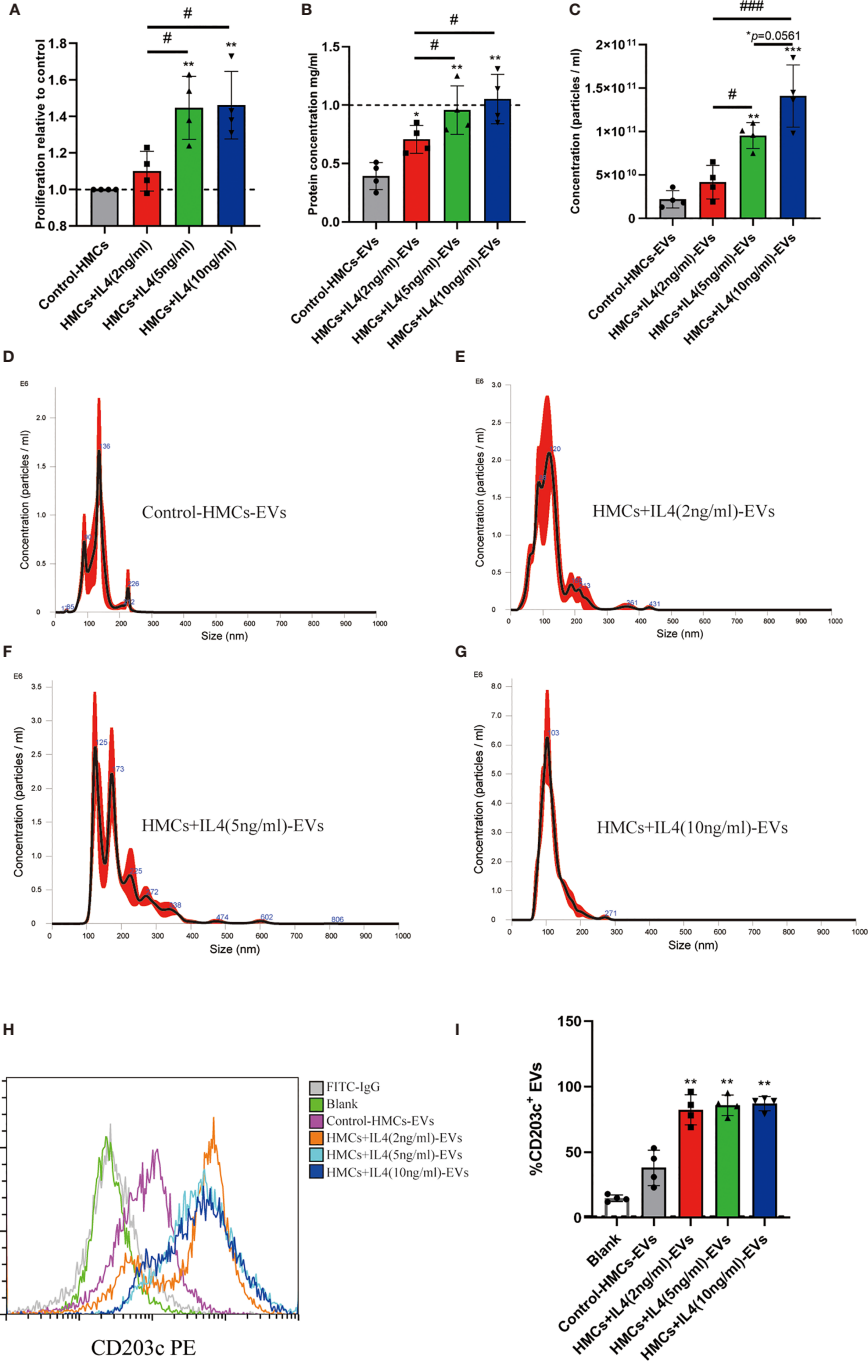
Proliferation of HMCs and CD203c^+^-EVs production were promoted by IL4. HMCs were stimulated with differential concentration of IL4, and EVs were isolated using ultracentrifugation from culture supernatant for further analysis. **(A)** 2x10^6^ Human mast cells were seeded in T75 flask with serum free x-vivo 15 medium. After HMCs reached 70%-80% confluence, changed medium supplied with IL4 2ng/ml, 5ng/ml and 10ng/ml. After 24h incubation, proliferation of HMCs was determined by a BrdU incorporation assay. **(B)** After pelleted EVs from conditional supernatant of HMCs, the concentration of EVs in different groups was determined by a BCA assay. **(C–G)** The concentration and size distribution of EVs were determined by NTA test. The production of EVs was significantly increase in presence of IL4(5ng/ml and 10ng.ml). **(H, I)** The percentage of CD203c^+^-EVs in each group using flow cytometry, which showed that the percentage of CD203c^+^-EVs increased with increasing IL4 concentrations. All values represent the mean ± standard deviation. Difference to control: *p < 0,05; **p < 0,01; ^#^Difference between groups: ^#^ *p*< 0,05; ^###^ *p* < 0,001; 1 way ANOVA with Newman-Keuls Multiple Comparison Test; n = 4.

Previous reports have shown that IL4 promoted HMCs degranulation, cytokine production and FcϵRI expression (32–34). In this study, HMCs were stimulated with differential concentration of IL4. After 24h incubation, proliferation of HMCs was promoted in 5ng/ml and 10ng/ml groups (**Figure 4A**). However, there was no significant difference between the 5ng/ml and 10ng/ml groups, probably due to the fact that IL4 receptors on the surface of HMCs were saturated. After pelleted EVs from conditional supernatant of HMCs, the concentration of EVs was determined by a BCA assay and NTA. As shown in **Figures 4B–G**, the production of EVs was significantly increase in presence of IL4, this should be the result of elevated proliferation of HMCs. We identified the percentage of CD203c^+^-EVs in each group using flow cytometry, our results showed that the percentage of CD203c^+^-EVs increased with increasing IL4 concentrations (**Figures 4H, I**). Our results suggested that the level of CD203c^+^-EVs increased as HMCs were activated.

### CD203c^+^-EVs Derived From Basophils Were Significantly Increased in HR Patients

**Figure 5:**
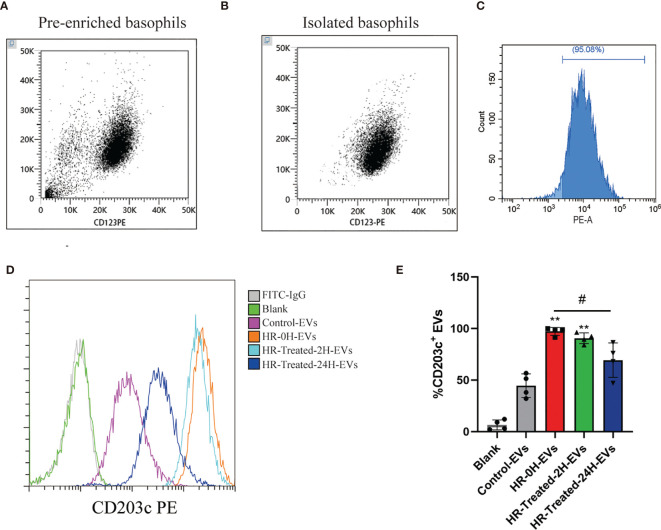
CD203c^+^-EVs derived from basophils were significantly increased in HR patients. Basophils from control and HR patients were isolated by negative selection method. Non-basophil cells in PBMCs were labeled with biotin-conjugated antibodies(CD3, CD4, CD7, CD14, CD15,CD16, CD36, CD45RA, HLA-DR and CD235a) and anti-biotin microbeads, then non-basophils were depleted by a BeyoMag™ separator.Then the pre-enriched basophils were labeled with CD123, and eluted from column after magnetic separation. After incubation using serum-free medium, EVs were isolated using ultracentrifugation from culture supernatant for further analysis. **(A–C)** The purity of pre-enriched basophils and isolated basophils were identified using flow cytometry, which expressed CD123 over 95%. **(D, E)** The percentage of CD203c^+^-EVs in control and HR groups were determined by flow cytometry. The percentage of CD203c^+^-EVs increased in HR-0H and HR-treated-2H group, which decreased after treatment in HR-treated-24H group. All values represent the mean ± standard deviation. Difference to control: **p < 0,01; ^#^Difference between groups: ^#^ *p* < 0,05; 1 way ANOVA with Newman-Keuls Multiple Comparison Test; n = 4.

Basophils were isolated by negative selection method. We identified the purity of basophils using flow cytometry, which expressed CD123 above 95% (**Figures 5A** **–C**). Basophils were seeded in T75 flask with serum free x-vivo 15 medium (Lonza). After 48h incubation, conditional culture supernatant was collected for EVs isolation using ultracentrifugation. The percentage of CD203c^+^-EVs in control and HR groups were determined by flow cytometry. As shown in **Figures 5D, E**, the percentage of CD203c^+^-EVs increased in HR-0H and HR-treated-2H group, which decreased after treatment in HR-treated-24H group. These results indicated that the level of CD203c^+^-EVs could monitor the activation of basophils in HR.

### HR Increased the Production of Histamine and Tryptase Levels and Expression of Inflammatory Mediators

Mast cell/basophil are activated when FcϵRI are cross-linked by the antigen and IgE antibodies, and then release of chemical mediators is followed by secretion of multiple cytokines, which induce the immediate-phase of allergic responses (35). Serum histamine and tryptase levels were determined using an ELISA kit and gene expression of inflammatory mediators (*IL1B*, *IL4*, *IL13*, *IL33*) was examined using real-time RT -PCR analysis. The concentrations of histamine and tryptase in serum increased significantly in the HR -0H and HR -treated-2H groups, which decreased in the HR -treated-24H groups (**Figures S2A, B**). Moreover, the gene expression of *IL1B*, *IL4*, *IL13*, *IL33* was significantly promotedin HR -0H and HR - treated-2H groups(**Figures S2C–F**).

### Protein Expression and Concentration of CD203c^+^-EVs Positively Correlates With Inflammatory Mediators

**Figure 6:**
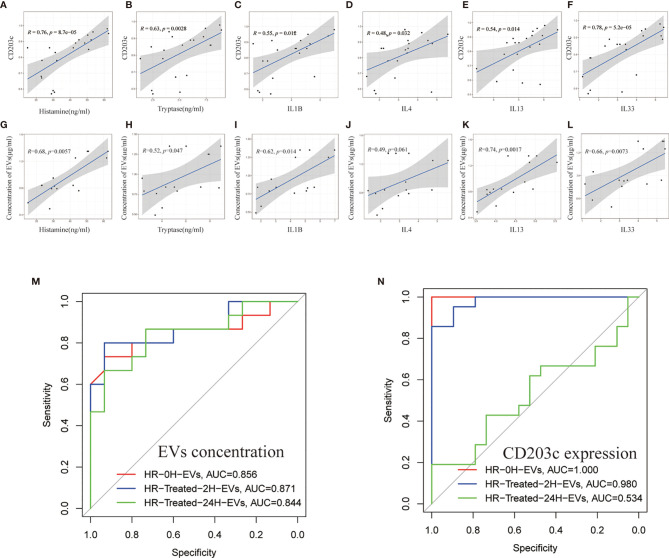
The protein expression and concentration of CD203c^+^-EVs positively correlated with inflammatory mediators. **(A–F)** Correlation analysis between CD203c expression levels(flow cytometry) in EVs and inflammatory mediators in serum. R: Pearson correlation coefficient, Correlation coefficient |R|>0.5, *p* < 0.05 was considered statistically significant. **(G–L)** Correlation analysis between the concentration of EVs and inflammatory mediators. R: Pearson correlation coefficient, Correlation coefficient |R|>0.5, *p* < 0.05 was considered statistically significant. **(M, N)** ROC curve of EVs concentration and CD203c expression in different timepoint groups for predicting the diagnosis of HR.

To investigate the relationship between EVs and HR induced inflammatory mediators, Pearson correlation coefficient analysis was used to reveal the correlation between EVs concentration/CD203c expression and inflammatory mediators. Our results showed that both EVs concentration and CD203c expression(based on the results of flow cytometry) were positively correlated with histamine, tryptase, *IL1B*, *IL4*, *IL13*, and *IL33* (**Figures 6A** **–L**).

Moreover, the receiver operating characteristic curve (ROC) of EVs concentration/CD203c expression was used to analyze the diagnostic ability of EVs. As shown in **Figures 6M, N**, CD203c^+^-EVs exhibited strong diagnostic ability for HR. These results indicated that the CD203c^+^-EVs from HR serum could be a potential marker for the diagnosis and prognosis of HR.

## Discussion

**Figure 7:**
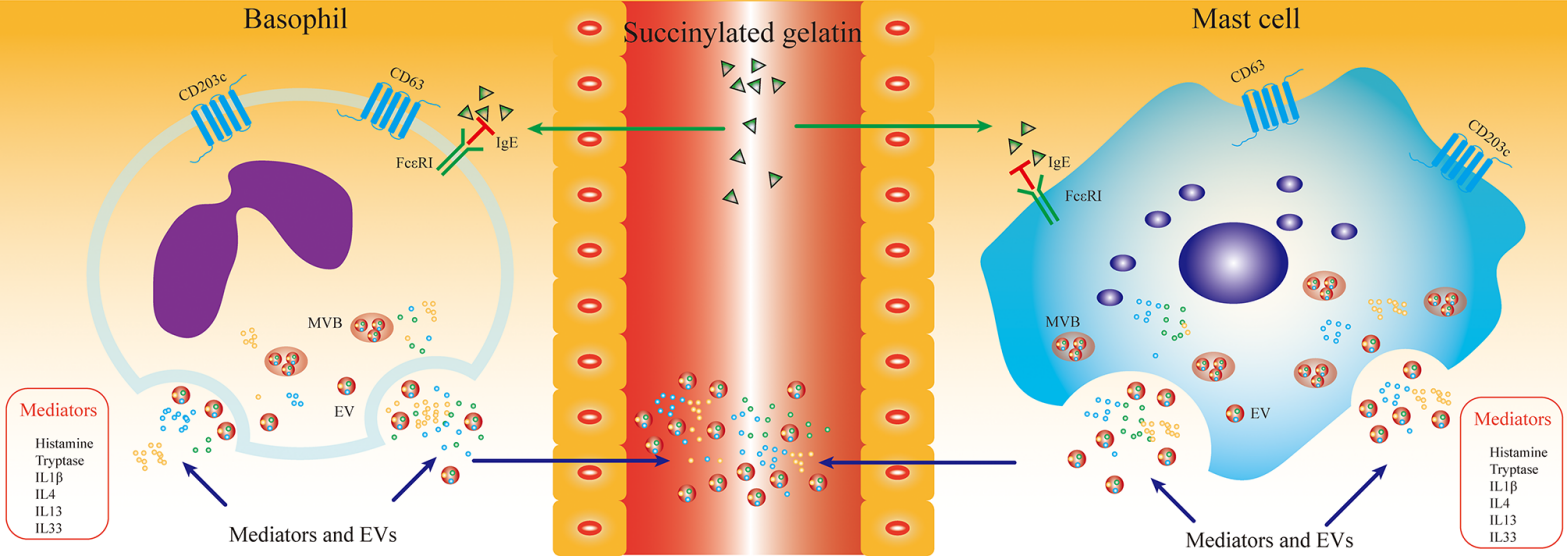
SG induced HR promoted the production of mast cell/basophil specific EVs. The aim of this study was to evaluate the diagnostic sensitivity of mast cell/basophil specific EVs in SG induced HR. HR triggered mast cell/basophil degranulation and activation, which leaded releasing EVs and inflammatory mediators into serum. Serum was obtained from SG induced HR patients at different time point. The mast cell/basophil specific EVs and inflammatory mediators in serum were determined and analyzed. EVs derived from HR groups enriched specific proteins (CD63, CD203c, FcϵRI and tryptase) of mast cell/basophil, which were related with the inflammatory levels induced by SG. We hypothesize that mast cell/basophil specific EVs could serves as a novel diagnostic and prognostic biomarker for SG induced HR. Abbreviations: SG, Succinylated gelatin; MVB, multivesicular body.

Previous studies have shown that inflammatory mediators associated with degranulation and activation of mast cells and basophils are altered in the plasma/serum of HR patients and can be used as molecular markers for the diagnosis of HR (36, 37). However, the sensitivity and specificity of these mediators for HR diagnosis and prediction of prognosis in plasma/serum were not satisfactory. Histamine and tryptase are preformed molecules produced and stored in mast cells and basophils and serve as markers for mast cell and basophil activation. However, measuring plasma levels of histamine can be difficult due to its short half-life, which returns to baseline within 15-30 minutes after the onset of activation (38). Furthermore, there is no consensus on the minimal increase in serum tryptase that indicates a “mast cell activation-related” event (39). In the present study, we first investigated CD203c^+^-EVs as a specific membrane-associated activation marker of mast cells and basophils to diagnose and predict the prognosis of HR, which was satisfactory. Experimental outline and hypothesis of this study are shown in **Figure 7**.

There are few studies of EVs in serum from HR. We pelleted EVs from the serum of HR patients and found that the concentration of EVs was significantly increased in HR samples and decreased after treatment, but no obvious difference in size. CD63 and CD203c has been identified as activation markers of mast cells/basophils, we first time investigated the CD203c express level in EVs from HR patients. Our results showed that the expression level of CD63 and CD203c in EVs was significantly upregulated in HR samples, but no significant difference in CD9 and CD81. These results suggest that the increased CD63^+^- EVs and CD203^+^- EVs are related to mast cell and basophil activation. It has been reported that CD63 and CD203^+^-EVs were upregulated by FcϵRI-mediated responses (40). Similarly, our results showed that the expression level of CD63 and CD203 was increased in the serum of HR samples, but no significant difference. We assumed that CD203c in EVs could serve as biomarkers for HR.

The mast cell/basophil-specific proteins, CD203c, and tryptase have been shown to be associated with mast cell and basophil degranulation and activation (10, 41, 42). In this study, the expression levels of FcϵRI, CD203c, CD63, and tryptase were significantly upregulated in EVs from HR samples, which showed that the expression levels of FcϵRI, CD203c and CD63 were all related with mast cells/basophils activation. Interestingly, FcϵRI, CD203c and CD63 were also detected in control EVs, which could be due to the accumulation of membrane proteins when EVs are released from mast cells/basophils. However, CD63 could be found in EVs derived from most kinds of cells, tissues and biofluid, moreover, FcϵRI was found not only on the surface of mast cells/basophils, but also eosinophils (43). In the present study, CD203c in EVs was selected to be a biomarker of mast cells/basophils activation for further analysis. Consistent with our hypotheses, the CD203c level and concentration of EVs were increased in untreated patients (HR), whereas they decreased after treatment, which was due to the fact that SG induced degranulation and activation of mast cells/basophils were inhibited by treatment. Mast cells/basophils activation markers CD203c were validated by flow cytometry, which was useful for monitoring allergen-specific mast cell/basophil activation (9). In the present study, flow cytometry results showed that the expression of CD203c on the surface of EVs was significantly overexpressed at the onset of HR. This suggests that CD203c ^+^-EVs may function as a novel mast cell/basophil-specific biomarker of HR.

In order to validate the specificity of CD203c^+^-EVs in HR, we first time isolated basophils from PBMCs of HR patients, and assessed the basophils derived EVs *in vitro*. We found that the level of CD203c^+^-EVs was significantly upregulated in HR samples. This result suggested that CD203c^+^-EVs could serve as a stable biomarker to monitor the activation of basophils, although we did not induce basophil activation during culture. This observation is consistent with previous studies (44, 45), which suggested that allergen-induced changes in CD203c expression of mast cells/basophils were not detected after 4 months of sublingual immunotherapy. Previous studies reported that IL4 could promoted degranulation of mast cells during IgE-mediated HR (46, 47). In our study, we stimulated HMCs with differential concentration of IL4, we found that IL4 increased the proliferation of HMCs and the production of EVs. Further analysis using flow cytometry proved that the percentage of CD203c^+^-EVs was increased in presence of IL4 (5ng/ml and 10ng/ml). In the present study, we found an interesting result that CD203c expression was detected in control patient EVs, which probably due to EVs enriched CD203c during the release procedure from mast cells/basophils. These results suggested that the level of CD203c^+^-EVs was associated with of mast cells/basophils activation.

Histamine, tryptase and inflammatory mediators (*IL1B*, *IL4*, *IL13*, *IL33*, etc.) are known to be released by mast cells and basophils during the progress of HR (48–50). The level of histamine, tryptase and inflammatory mediators in serum(without PBMCs isolation) of HR samples was determined. In accordance with previous studies, the results of this study showed that the concentration of histamine and tryptase and the gene expression of *IL1B*, *IL4*, *IL13*, *IL33* significantly increased in the serum of HR-0H samples, and decreased after treatment, that was because glucocorticoids suppressed the expression of inflammatory genes through inhibiting the transcription of AP-1 and NF-κB (51). We also performed correlation analysis between CD203c^+^-EVs and inflammatory mediators. The results showed that the concentration of EVs and the expression of CD203c were positively correlated with the concentration of histamine and tryptase and the gene expression of *IL1B*, *IL4*, *IL13*, *IL33*, which means that the concentration of CD203c^+^- EV as a method to indicate the level of the inflammatory mediators in HR. Moreover, the ROC curve of EVs concentration and CD203c expression suggested that CD203c^+^- EVs had a strong diagnostic ability for HR.

It has been reported that the diagnosis of immediate hypersensitivity starts with a thorough clinical history and physical examination and is confirmed by allergen extraction skin prick and allergen serum level of IgE (sIgE) measurement (52, 53). However, there are significant differences between clinical presentation (including skin prick tests) and sIgE measurement. For example, the predictive utility of serologic measurements of sIgE is not satisfactory for predicting food and drug allergies and asthma (52). Several studies have assessed the expression of various mast cells/basophils surface structures in an effort to develop new diagnostic and prognostic indicators for allergies (45, 54, 55). Furthermore, CD203c expression on mast cells/basophils has been used to diagnose different types of allergic diseases (8, 31, 56, 57). However, it is not easy to perform mast cell/basophil studies in the clinical setting because of the limited and complex procedures for mast cell/basophil isolation. It’s promising to identify practical biomarkers of mast cells/basophils activation *in vitro*. Here, we developed CD203^+^-EVs, which derived from HR serum present specific marker of mast cells and basophils which are stable and easily handled. In conclusion, the current study demonstrated that serum CD203c^+^-EVs exhibited high sensitivity and accuracy in diagnosing and predicting the prognosis of HR, which could serve as a novel diagnostic and prognostic biomarker for HR.

## Data Availability Statement

The original contributions presented in the study are included in the article/**Supplementary Material**. Further inquiries can be directed to the corresponding authors.

## Author Contributions

ZQ: sample collection, methodology, experiments design, validation, formal analysis, investigation, data curation, writing (original draft, review, and editing). QX and HW: conceptualization, writing (review and editing). BC: methodology, the establishment of EV isolation, and NTA analysis. QHX: reviewing, and writing; J-JY: conceptualization, writing (Review and editing), project administration, and funding acquisition. All authors contributed to the article and approved the submitted version.

## Funding

This study was supported by the National Natural Science Foundation of China (81971020).

## Conflict of Interest

The authors declare that the research was conducted in the absence of any commercial or financial relationships that could be construed as a potential conflict of interest.

## Publisher’s Note

All claims expressed in this article are solely those of the authors and do not necessarily represent those of their affiliated organizations, or those of the publisher, the editors and the reviewers. Any product that may be evaluated in this article, or claim that may be made by its manufacturer, is not guaranteed or endorsed by the publisher.

## Supplementary Material

Supplementary Figure 1 The expression level of CD203c in EVs derived from serum of Neuromuscular Blocking Agents, Succinylated Gelatin and Antibiotics induced HR. To evaluate the CD203c+EVs level in different drugs induced HR. EVs were isolated from serum of Neuromuscular Blocking Agents, Succinylated Gelatin and Antibiotics induced HR patients. **(A, B)** Quantitation of the percentage of CD203c+-EVs in each group using flow cytometry. The Control-EVs group is defined as control. All values represent the mean ± standard deviation. Difference to control: *p < 0,05; **p < 0,01; 1 way ANOVA with Newman-Keuls Multiple Comparison Test; n = 5.

Supplementary Figure 2 Serum mediators changes at different timepoints after treatment of HR An ELISA assay and real-time RT-PCR were performed to evaluate the inflammatory levels of HR serum induced by SG at different time point. **(A, B)** An ELISA assay was employed to evaluate the histamine and tryptase levels in serum. Histamine and tryptase concentrations in serum increased at the beginning of HR(0H) and then decreased at 2 and 24 hours after treatment, respectively. **(C–F)** The gene expression of inflammatory mediators(IL1B, IL4, IL13, IL33) was determined with real-time RT-PCR analysis. The gene expression of IL1B, IL4, IL13 and IL33 was significantly upregulated onset of HR and decreased at 2h after treatment. All values represent the mean ± standard deviation. Difference to control: *p < 0,05; **p < 0,01; ***p < 0,001; ****p < 0,0001; #Difference between groups: ^#^p < 0,05; ^##^p < 0,01; ^###^p < 0,001; ^####^p < 0,0001;1 way ANOVA with Newman-Keuls Multiple Comparison Test; n = 15.
