# Supplementary figures and images for: Serum CD203c+ Extracellular Vesicle Serves as a Novel Diagnostic and Prognostic Biomarker for Succinylated Gelatin Induced Perioperative Hypersensitive Reaction
Source: Front Immunol. 2021 Sep 28;12:732209. doi: 10.3389/fimmu.2021.732209 (PMC8505883; doi:10.3389/fimmu.2021.732209)

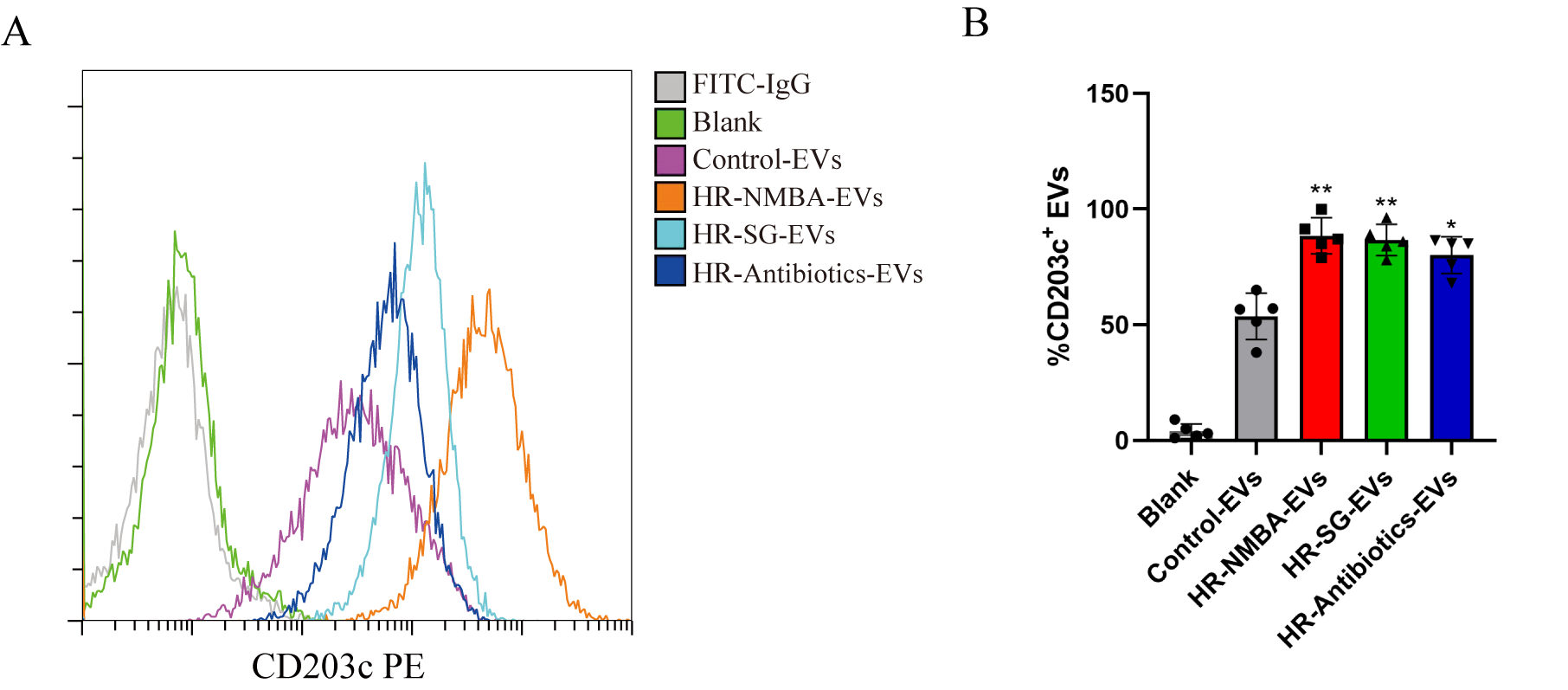

Supplement: Supplementary Figure 1 — The expression level of CD203c in EVs derived from serum of Neuromuscular Blocking Agents, Succinylated Gelatin and Antibiotics induced HR. To evaluate the CD203c+EVs level in different drugs induced HR. EVs were isolated from serum of Neuromuscular Blocking Agents, Succinylated Gelatin and Antibiotics induced HR patients. (A, B) Quantitation of the percentage of CD203c+-EVs in each group using flow cytometry. The Control-EVs group is defined as control. All values represent the mean ± standard deviation. Difference to control: *p < 0,05; **p < 0,01; 1 way ANOVA with Newman-Keuls Multiple Comparison Test; n = 5. [file Image_1.tif]

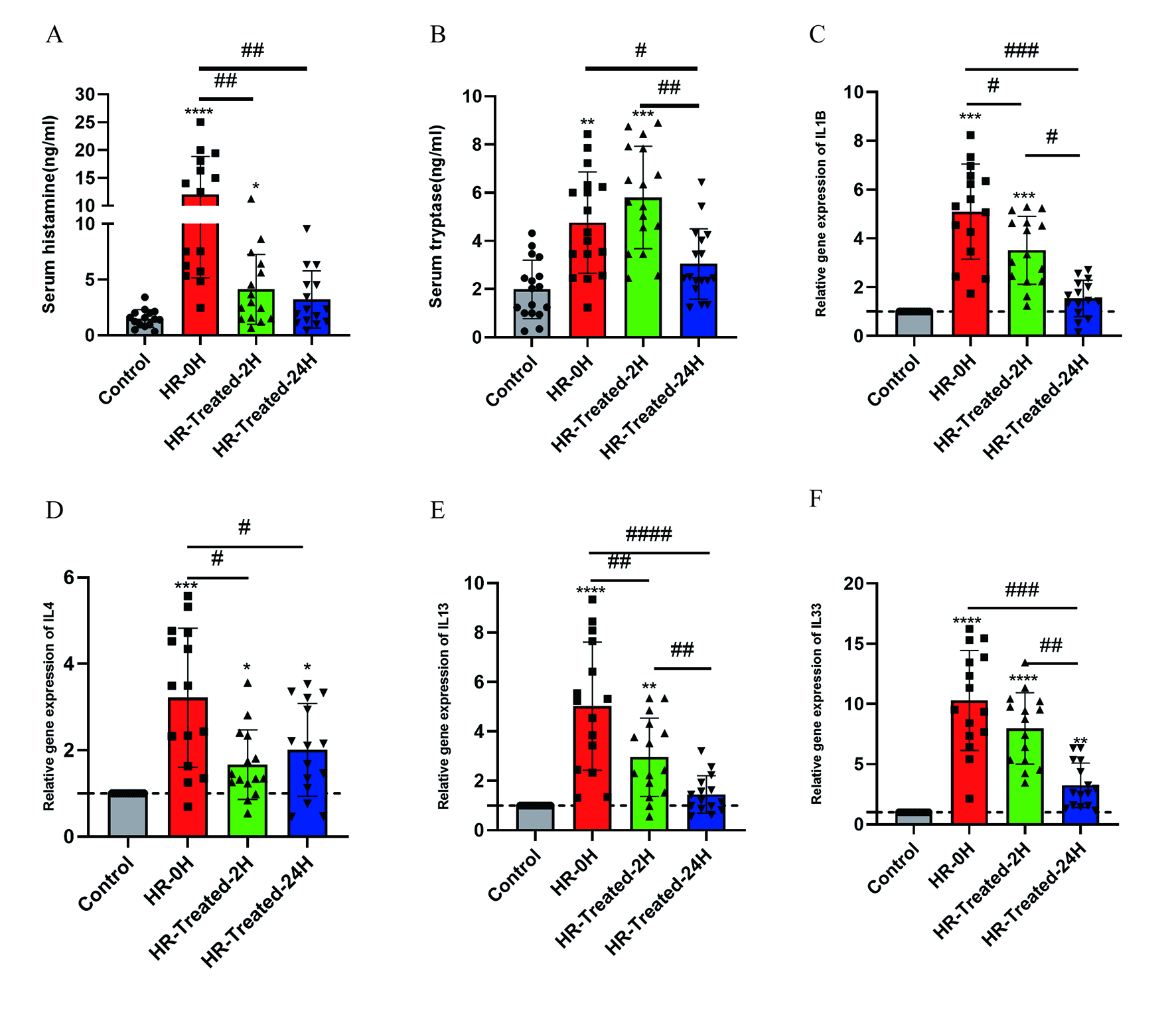

Supplement: Supplementary Figure 2 — Serum mediators changes at different timepoints after treatment of HR An ELISA assay and real-time RT-PCR were performed to evaluate the inflammatory levels of HR serum induced by SG at different time point. (A, B) An ELISA assay was employed to evaluate the histamine and tryptase levels in serum. Histamine and tryptase concentrations in serum increased at the beginning of HR(0H) and then decreased at 2 and 24 hours after treatment, respectively. (C–F) The gene expression of inflammatory mediators(IL1B, IL4, IL13, IL33) was determined with real-time RT-PCR analysis. The gene expression of IL1B, IL4, IL13 and IL33 was significantly upregulated onset of HR and decreased at 2h after treatment. All values represent the mean ± standard deviation. Difference to control: *p < 0,05; **p < 0,01; ***p < 0,001; ****p < 0,0001; #Difference between groups: #p < 0,05; ##p < 0,01; ###p < 0,001; ####p < 0,0001;1 way ANOVA with Newman-Keuls Multiple Comparison Test; n = 15. [file Image_2.tif]
